# A Synthetic Population for Modelling the Dynamics of Infectious Disease Transmission in American Samoa

**DOI:** 10.1038/s41598-017-17093-8

**Published:** 2017-12-01

**Authors:** Zhijing Xu, Kathryn Glass, Colleen L. Lau, Nicholas Geard, Patricia Graves, Archie Clements

**Affiliations:** 1 Research School of Population Health, Australian National University, Canberra, Australia; 2 School of Computing and Information Systems, University of Melbourne, Melbourne, Australia; 3 Melbourne School of Population and Global Health, University of Melbourne, Melbourne, Australia; 4 College of Public Health, Medical and Veterinary Sciences, Division of Tropical Health and Medicine, James Cook University, Cairns, Australia

## Abstract

Agent-based modelling is a useful approach for capturing heterogeneity in disease transmission. In this study, a synthetic population was developed for American Samoa using an iterative approach based on population census, questionnaire survey and land use data. The population will be used as the basis for a new agent-based model, intended specifically to fill the knowledge gaps about lymphatic filariasis transmission and elimination, but also to be readily adaptable to model other infectious diseases. The synthetic population was characterized by the statistically realistic population and household structure, and high-resolution geographic locations of households. The population was simulated over 40 years from 2010 to 2050. The simulated population was compared to estimates and projections of the U.S. Census Bureau. The results showed the total population would continuously decrease due to the observed large number of emigrants. Population ageing was observed, which was consistent with the latest two population censuses and the Bureau’s projections. The sex ratios by age groups were analysed and indicated an increase in the proportion of males in age groups 0–14 and 15–64. The household size followed a Gaussian distribution with an average size of around 5.0 throughout the simulation, slightly less than the initial average size 5.6.

## Introduction

Agent-based models are increasingly used to investigate the processes, mechanisms and behaviours of many complex social systems due to their ability to capture the nonlinear dynamics of social interactions. For infectious diseases, agent-based modelling has demonstrated considerable value for informing public health policies aimed at preparation for or response to epidemics^1–5^, including the 2014–2016 Ebola outbreak in West Africa^6–8^. Recent studies on transport simulation and disease modelling have highlighted the value of synthetic populations^9–13^, especially when heterogeneities in population mixing play a crucial role in disease transmission^14^, or when disease incidence or risks of infection vary significantly between subgroups, such as age groups^15–17^. The finding of highly variant prevalence of lymphatic filariasis across gender and age groups^18^ further highlights the importance of demographics in transmission dynamics. For American Samoa, the age structure is distorted by the large emigration to the United States and immigration from Western Samoa, leading to the incapability of present models on long-term transmission dynamics^16,17^.

Synthetic populations can be categorised according to the level of detail they capture about real populations. One important distinction is whether or not they explicitly represent the geographic locations of individuals. Spatial models of disease transmission are an important minority. High-resolution geographic locations of human individuals are rarely included in models for diseases transmitted by person-to-person contact^11,19–21^. Spatial population data in these studies are primarily used to map epidemics rather than project the risk of infection at the individual level. However, high-resolution geographic locations of human individuals are more critical for infectious diseases that demonstrate significant heterogeneity at small spatial scales or show household-level clustering. Examples include vector-borne diseases, where vector abundance can vary dramatically with environmental settings; and water-borne diseases such as leptospirosis, where household-level factors like flooding risk play an important role in transmission. Further, diseases that are not highly infectious are more likely to show spatial clustering because transmission is unlikely unless there is repeated or prolonged exposure. For example, lymphatic filariasis (LF) is not highly infectious, and in the disease elimination setting, residual hotspots of transmission can be highly focal^18^. In this study, we built a synthetic population as the basis of an agent-based model to improve our understanding of dynamics of LF transmission in American Samoa, but the synthetic population can also be used to model other infectious diseases, particularly those with significant spatial heterogeneity.

Lymphatic filariasis (LF) is a parasitic worm infection, transmitted from person to person by mosquito bites. The adult worms live and mate in the human lymphatic system, and can be reproductively active for 5–8 years^22^, producing millions of microfilariae that circulate in the blood and infect the mosquito during a biting event. The ingested microfilariae grow and develop into L3 larvae in the mosquito. The L3 larvae can then pass from the mosquito to a human in subsequent biting events, and grow into adult worms in six months or more^23^. The adult worm has a mean life span of 10 years, with a maximum of 14 years^22^. A wide range of mosquitoes can transmit the parasite, depending on the geographic area. In American Samoa, the main vectors of LF are *Aedes* mosquitoes, which have a shorter average flight distance (no more than 150 m) than other species^24,25^. Filarial worms can only be transmitted to humans within the flight range of infectious mosquitoes, which means that people, rather than mosquitoes, are predominantly responsible for the movement of filarial worms within and between communities and countries. Clinical presentation of LF usually develops many years after infection, and LF laboratory diagnostic tests (microfilariae and antigen) are often negative by the time of clinical disease. Due to the extremely long life expectancy of adult filarial worms, the many years that people can be infectious for, and the long interval (years) between infection and clinical presentation, modelling the demographics over an extended period is of critical importance for investigating the transmission dynamics of LF.

Initiated in 1999, the Pacific Programme for Elimination of Lymphatic Filariasis (PacELF) aims to eliminate LF in 22 Pacific Island countries and territories (PICTs) by 2020 as part of the Global Programme to Eliminate LF (GPELF). Infection prevalence has been significantly reduced in PICTs, with considerable success achieved in American Samoa, where the antigen prevalence dropped from 16.50% in 1999 to 2.29% in 2007 after seven rounds of mass drug administration (MDA) from 2000 to 2006^26^. However, there are significant knowledge gaps about LF transmission dynamics, particularly at the end stages of elimination programs when prevalence has reached very low levels. Recent studies have raised concerns about the recommended target threshold (<1% antigenaemia) for post-MDA transmission assessment surveys (TAS)^27^, and it is unclear whether school-based surveys of young children provide an accurate reflection of infection rates in older age groups. Also unknown is the expected size of any residual hotspots of transmission, and the distance of influence of infected individuals on their near neighbours. Current mathematical and computational models of LF do not capture small-scale spatial heterogeneity of risk factors^16,17^, restricting our understanding of LF dynamics at low prevalence. To accurately model spatial heterogeneity of LF transmission over extended periods of time, there is a need for models of synthetic populations that incorporate reliable high-resolution geographic data.

In this study, we use an iterative approach^28^ to generate a synthetic population for American Samoa based on 2010 census data^29^, household data from a seroprevalence study conducted in 2010^18,27^ and high-resolution geo-referenced land use data^30^. Our synthetic population was developed to capture the hierarchical social structure (household, village and county) using real geographic locations of buildings. Individuals are represented as fully dynamic agents and are allocated into households with particular roles (householder, partner, children, other relatives or nonrelatives) according to their relationships with the householder. Major dynamic processes such as birth, death, couple formation/separation, and migration were modelled, allowing for the simulation of the population evolution over an extended time frame. The synthetic population aimed to provide a statistically realistic population with reliable long-term demographic evolution that is suitable for modelling LF. The demographic and geographic data sources, together with methods used to generate and simulate the evolution of the synthetic population were described in the following section. Model output was then compared to estimates and projections from the U.S. Census Bureau and observed differences were evaluated and discussed.

## Results

### Static Population

**Figure 1:**
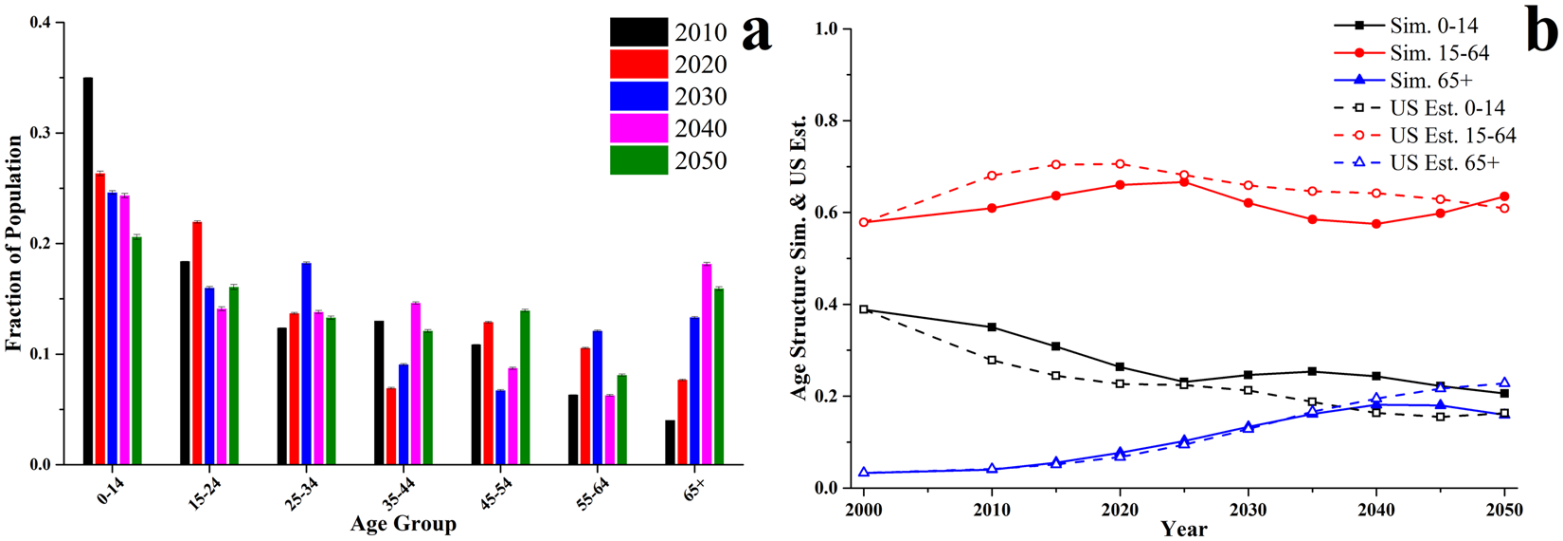
Population in American Samoa: (**a**) simulated population by age group; (**b**) simulated (sim.) and the Bureau projected (US Est.) population by broad age group. Error bars in (**a**) indicate standard deviation based on 20 simulations. The simulation started in 2010, while in (**b**) the data points in 2000 were also added to offer an explicit comparison with the estimates and projections from the Bureau.

The age structure of the static synthetic population was consistent with the 2010 census population in American Samoa (black bars in Fig. 1(a)). The synthetic population by broad age groups (0–14, 15–64 and 65+) was also compared to projections from the Bureau, which were based on the 2000 census population and adjusted by the 2010 census population. Figure 1(b) indicates the Bureau has significantly underestimated the population in age group 0–14 (27.8% vs 35.0% in the 2010 census population) and overestimated the population in age group 15–64 (68.0% vs 61.0%), a combined effect of underestimated fertility rates and mortality rates. Data in the statistical yearbook 2004–2010 indicated that there were 371 more newborns and 219 more deaths than was projected by the Bureau. Net emigration also deviated from the projections. In 2006–2010, the actual number of net emigrants was 11,297 (4,413 more than projected).

**Figure 2:**
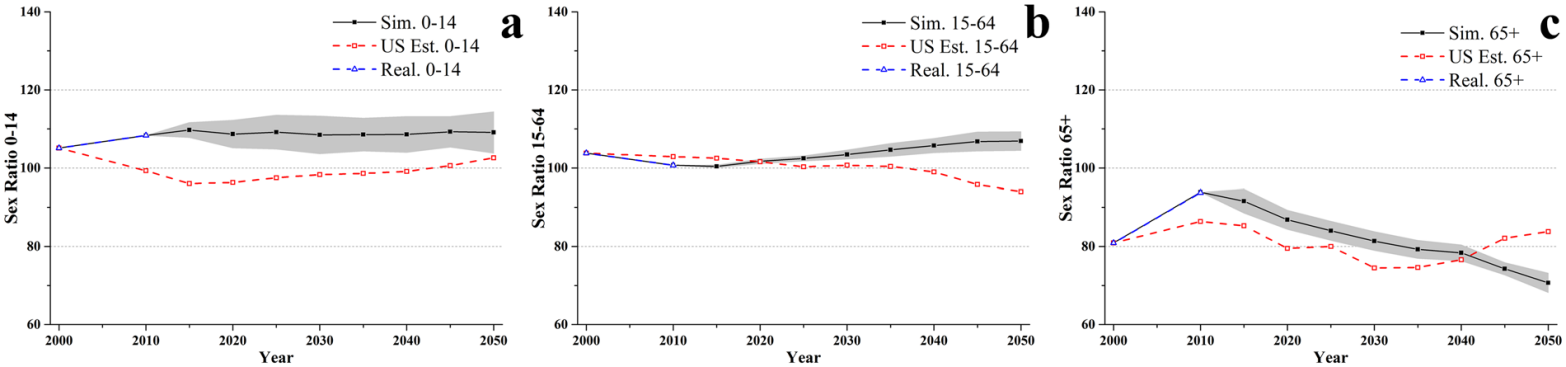
Sex ratio (males/100 females) by broad age group (**a**) 0–14, (**b**) 15–64, and (**c**) 65+: realistic in the population census (Real., blue open triangles); simulated (Sim., black solid squares); and the Bureau projected (US Est., red open squares). Grey area indicates the 95% confidence intervals based on 20 simulations.

The sex ratio by broad age group in the synthetic population indicated a good match with that in the 2010 census population (see Fig. 2). The comparison with the Bureau’s projections showed the Bureau had underestimated the proportion of males in the population aged 0–14 (Fig. 2(a)) and 65+ (Fig. 2(c)), and overestimated the proportion of males in age group 15–64 (Fig. 2(b)). The differences in the sex ratios from the Bureau’s projections have impacts far beyond the sex ratios themselves, especially in the childbearing age group 15–49. An overestimation of males in the age group implies an underestimation of the number of newborns, even if the total population in the age group matches the population census.

### Households

**Figure 3:**
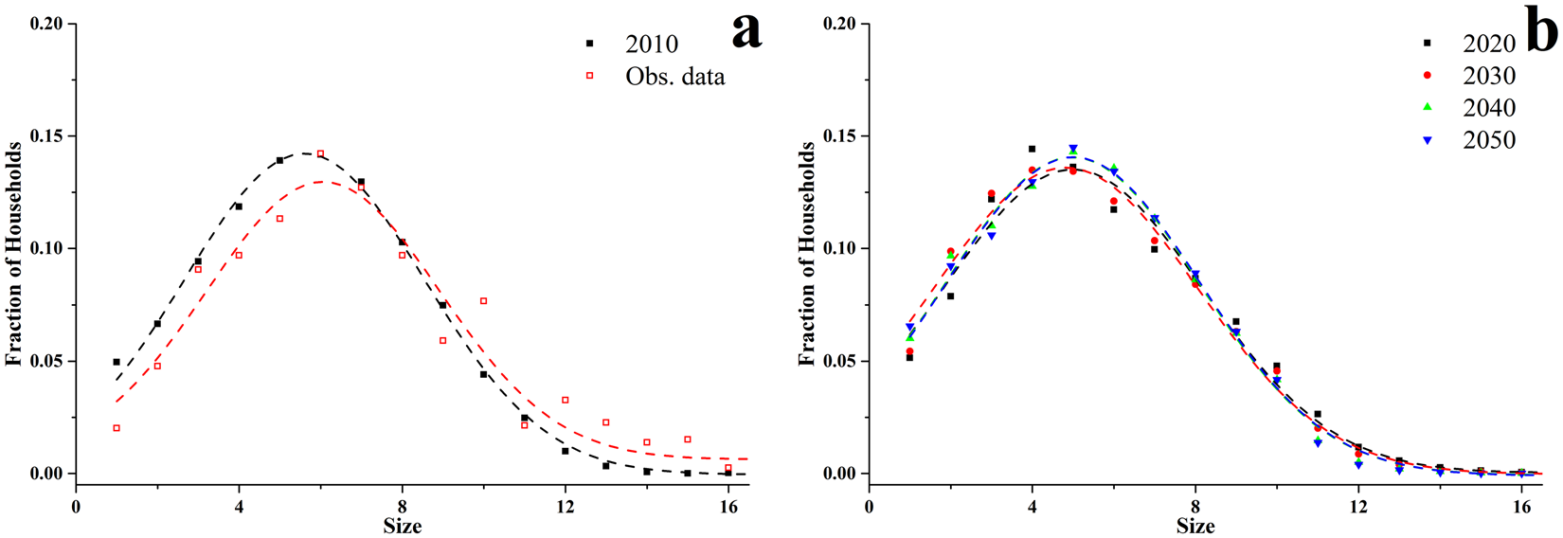
Household size distribution: (**a**) observed in the survey (Obs. data) and synthetic households (2010); (**b**) simulated households in 2020–2050. Dashed lines are the Gaussian fitting with parameters (μ, σ, Adj. R^2^): Obs. data-(6.06, 2.86, 0.944); 2010-(5.60, 2.95, 0.998); 2020-(4.99, 3.19, 0.983); 2030-(4.82, 3.24, 0.992); 2040-(4.98, 3.10, 0.996); 2050-(5.00, 3.11, 0.995).

In 2010, American Samoa had 9,688 occupied households, of which 288 were in the excluded villages of Swains Island and Rose Atoll. Consequently, there were initially 9,400 households in the synthetic population. The simulated household size followed a Gaussian distribution with parameters *μ* = 5.60 and *σ* = 2.95 (*R* ^2^ = 0.998) (black dashed line in Fig. 3(a)). The average size of the simulated households was consistent with the observed size in the 2010 survey (red dashed line in Figure 3(a), *μ* = 6.06 and *σ* = 2.86).

**Figure 4:**
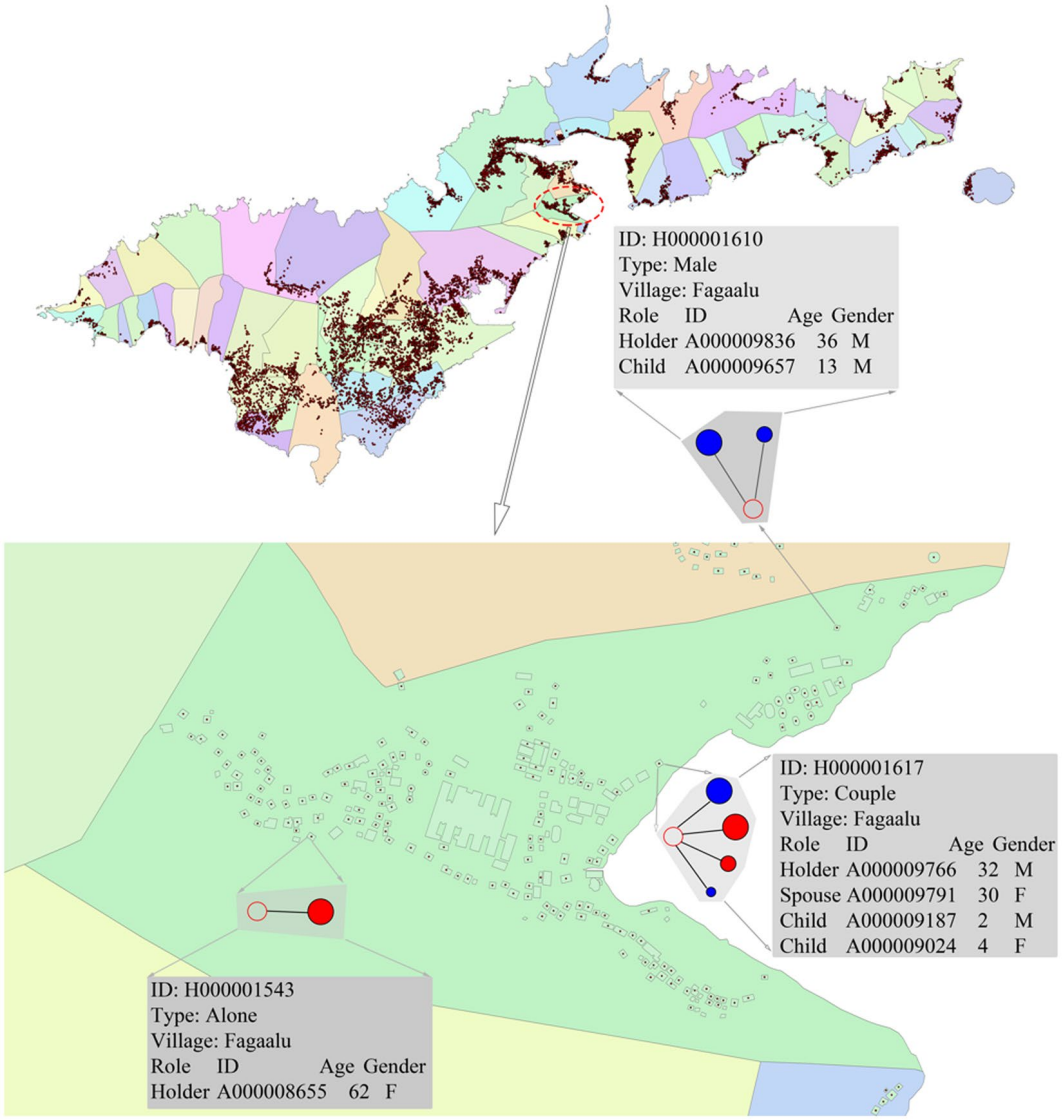
Simulated household locations in American Samoa. Multi-coloured polygons represent the villages. Households are represented by minor dots on the map. Each household is located into a residential building as indicated in the enlarged area (village Faga’alu). Household information is recorded in the simulation. Map is generated with ArcGIS Desktop 10.4.1^39^: http://desktop.arcgis.com/en/.

Following generation of residential buildings, our model contained 12,494 residential buildings for American Samoa in total. Each household was allocated a random residential building, ensuring the living space per capita was plausible. Households are indicated as minor dots in Fig. 4, in which the enlarged area is an illustration of the village of Faga’alu. The model contains 9,400 households, with 3,094 vacant residential buildings available for extra households when simulating the evolving synthetic population.

### Population Evolution

**Figure 5:**
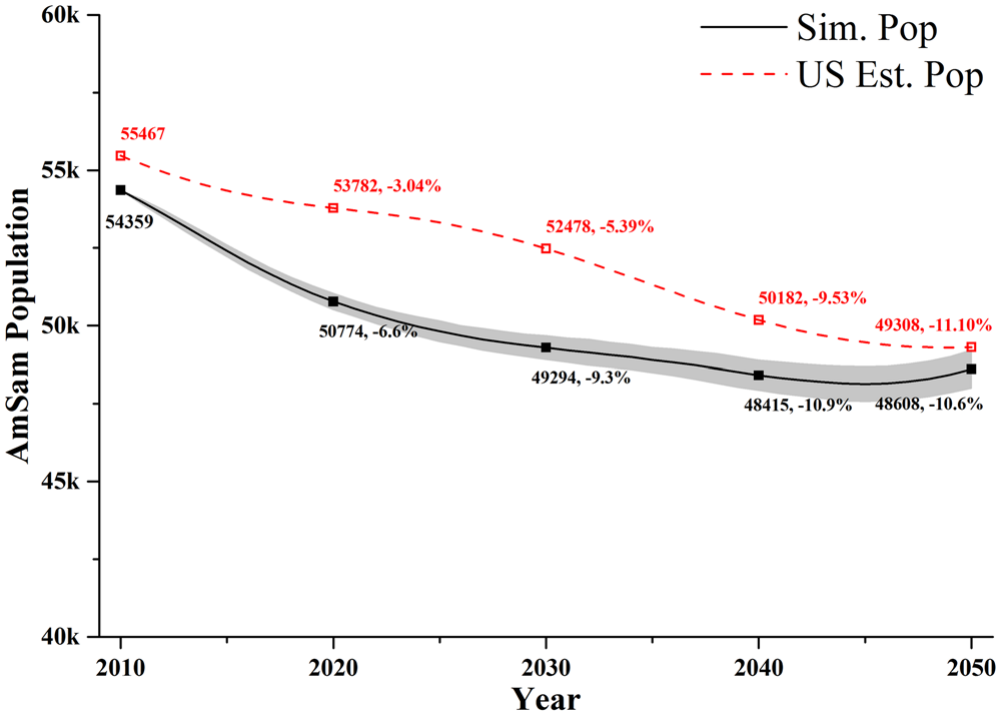
Total population 2010–2050 in American Samoa: simulated (Sim. pop) and the Bureau projected (US Est. pop). Data points on the lines indicate the total population at that time and the change compared to the initial population in 2010. Grey area indicates the 95% confidence intervals based on 20 simulations.

The synthetic population was simulated over a 40-year period, beginning in 2010. The population structure over 2010–2050 was compared to estimates and projections from the Bureau to evaluate its reasonableness and reliability. The modelled population of American Samoa was found to decrease by approximately 10.9% in the first 30 years due to the large net emigration, and then increase slightly in the following 10 years (Fig. 5). The increase in the population over 2040–2050 was attributed to the significant decrease in the number of net emigrants in the estimates and projections of the Bureau. Generally, the population was expected to decrease more rapidly in the first 20 years compared to the Bureau’s projections, and more slowly in the second 20 years (Fig. 5).

The mean age of the population increased over 2010–2050, with fewer children aged 0–14 and more individuals over 65+ (Fig. 1). As the large number of individuals aged 0–14 in 2010 reached age 25–34, the simulated population in age group 25–34 peaked around 2030. The simulated population aged 65+ in 2010–2040 was similar to the Bureau’s projections in Fig. 1(b). However, the simulated population aged 0–14 was always higher than the Bureau’s projections, and the simulated population aged 15–64 was always lower than the projections. In our simulation, the net emigration occurred primarily in the age group 25–34. The simulated population aged 0–14 would decrease if more individuals in this group were assumed to emigrate.

The simulated sex ratios (males per 100 females, *r*) by broad age groups were compared to the projections of the Bureau in Fig. 2. The sex ratio for individuals aged 65+ showed a similar trend to the Bureau’s projections in 2010–2040 (Fig. 2(c)). However, the sex ratios for those aged 0–14 (Fig. 2(a)) and 15–64 (Fig. 2(b)) were quite different to the Bureau’s projections, especially for ages 15–64. In Fig. 2(a), the sex ratio for 0–14 was simulated to be more than 100 (more males) in 2010–2050, which was higher than the Bureau’s projections (*r* < 100 in 2010–2040, more females). Furthermore, the simulated sex ratio was higher than that at the beginning of the simulation than in all subsequent years (2011–2050), which could be explained by the observed central rate of mortality in 1990, 2000 and 2011 (see Supplementary Fig. S2). Both the mortality rates for males and females aged 0–14 decreased from 2000 to 2010, however the decrease was more rapid for males, indicating the proportion of males in this group would increase in the future. In Fig. 2(b), the simulated sex ratio for 15–64 showed an opposite trend to that of the Bureau’s projections. The proportion of males in age group 15–64 was projected to increase from 2010 to 2050 in our simulation, while it decreased in the Bureau’s projections. A further inspection of the mortality rate justified the trend in our simulation. The Supplementary Fig. S2(b) indicated the mortality rate for males aged 15–64 continuously decreased from 1990 to 2011. By contrast, the mortality rate for females aged 15–64 increased in that period.

The household size followed a Gaussian distribution throughout the simulation with average size fluctuating around 5.0 (Fig. 3(b)), slightly less than the initial average size of 5.6 (Fig. 3(a)). A decreasing trend in the average household size was found in American Samoa in the census data from 7.2 in 1980 to 5.7 in 2010. The trend may continue in the next 40 years, as indicated in the simulation. However, the common extended family structure in American Samoa might maintain a high average household size. One of the possible reasons for the shrinking households is decreasing fertility. In the last two population censuses, the total fertility rate had decreased from 4.00 in the Population Census 2000 to 3.76 in the Population Census 2010. According to the Bureau’s projections, the total fertility rate will continuously decrease to 2.08 in 2050.

## Discussion

In this study, a synthetic population of American Samoa was constructed using an iterative approach based on population census, questionnaire survey and land use data. The model captured the age- and household structure of American Samoa, while simulation of the population evolution indicated a decreasing and ageing population. The simulation output was compared to estimates and projections of the U.S. Census Bureau. The simulated age structure and sex ratios by age groups indicated some discrepancies with the Bureau’s projections, which could be explained by observed fertility and mortality trends in recent Population Censuses in American Samoa. The household size followed a Gaussian distribution with average size around 5.0 throughout the simulation, slightly less than the initial average size of 5.6. The model considered the extended time frame (40 years) and high-resolution spatial distribution of the population, which makes it suitable for modelling LF and other infectious diseases with long incubation and long infectious periods, such as tuberculosis.

The age structure of the population was calibrated to the 2010 census population. The absence of data on household structure required us to make some assumptions when generating the synthetic population and modelling movements between households. The strategy developed in Section 4.3.3 was intended to minimize the uncertainties in modelling household dynamics. Parameters relating to movements between households were more speculative than other parameters. While we included many mechanisms driving these movements, we did not include the informal adoption of children, a very common practice in American Samoa, because no data were available. Households with more than five members accounted for about 75% of all households in American Samoa, among which the extremely large ones could consist of more than twenty members. The large household size may contribute to transmission of LF due to the relatively high risk of infection within the household. The large household size also increases the uncertainty in building households with no available data on the household structure. Further data relating to these components of the model would improve its accuracy and reliability. Households headed by same-sex couples were not included in our model due to a lack of data.

Natural population dynamics such as ageing, reproduction, marriage and divorce are simulated in most synthetic populations that extend over many years^10,14,31^. However, unlike these studies, the synthetic population in American Samoa was modelled as an open population with net loss of people due to net emigration. Both statistically realistic synthetic populations and population evolution have not been included in past LF modelling studies^32–34^ and LF dynamic models^16,17^, though they are of critical importance for modelling LF transmission dynamics and elimination. While we do not aim to interpret and understand the structural and dynamic evolution of populations (as in Geard *et al*.^31^), our objective is to develop a statistically realistic population underpinning further modelling studies of LF. Therefore, instead of updating the population with historical census data, the synthetic population in American Samoa was modelled with the latest available demographic data as well as projected fertility/mortality rates and net emigration assumptions. Besides the discussed combined effect of underestimated fertility rates and mortality rates in Section 2.1, net emigration could be another contributing factor to the Bureau’s deviations in age structure. Due to lack of details about how the Bureau developed projections of emigration for each age group, a complete analysis of the sources of deviation would be impossible. However, the Bureau’s underestimation of the population in age group 0–14 is likely to be partly due to overestimation of emigration in this age group. The differences in Fig. 4(b) are potentially linked to the approach used to determine net emigration in each age group, which highlight the importance of further exploration on age structure of the migration population.

Human activity patterns differ tremendously between day-time and night-time, as do the locations of the activities. While most individuals will be at their household locations during the night, many will go to work or school during the day. Modelling these locations is critical for capturing the diurnal variation in infection risk of vector-borne diseases. Although many mosquitoes are more likely to feed during night-time, *Aedes* mosquitoes in American Samoa can have different biting hours, including *Ae. polynesiensis* (day-biting), *Ae. samoanus* (night-biting), *Ae. tutuilae* (night-biting) and *Ae. upolensis* (day-biting)^27^. The next phase of our model development will be to add daily activities to the synthetic population with the aim of modelling LF transmission.

Our synthetic population is intended for modelling infectious diseases in American Samoa, with LF our initial disease focus. There have been numerous outbreaks of arboviruses in American Samoa (and other Pacific Islands) in recent years, including dengue, Chikungunya, and Zika, and we aim to build a synthetic population that is sufficiently flexible to be applied to understand arbovirus transmission and help inform control strategies for a range of pathogens. The model will also be suitable for infectious diseases with long incubation and long infectious periods, such as tuberculosis. The high-resolution spatial component of the model will be particularly valuable for diseases that show clustering within households or neighbourhoods. Furthermore, the synthetic population in this study could also be applied to investigate demographic changes, as demonstrated by the comparison of the simulation output and the projections of the US Census Bureau. To fully explore such demographic changes, fertility and mortality trends should be projected. To explore potential changes in household size, further data on changes in household structures would be required.

## Methods and Materials

### Data sources

**Figure 6:**
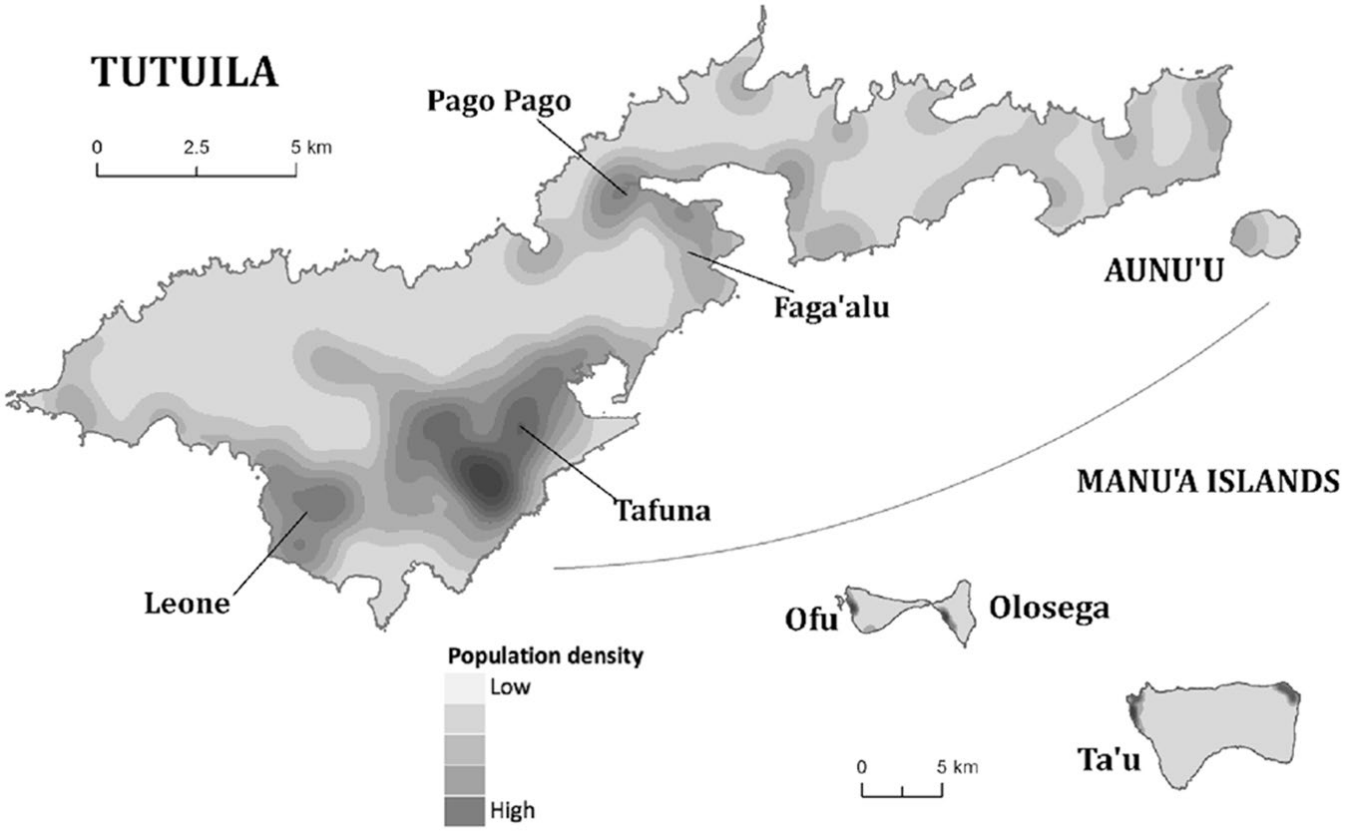
Islands and kernel smoothed population density in American Samoa (reproduced from ref.^18^). The populations in the districts are: Eastern District - 23,030; Western District - 31,329; Manu’a District - 1,143; Swains Island - 17. Total census population in 2010 is 55,519. The population in Tutuila and Aunu’u is 54,359 (97.9%).

American Samoa, located in Oceania, is a U.S. territory covering five main South Pacific islands (Tutuila, Aunu’u, Ofu, Olosega, and Tau, see Fig. 6) and two atolls (Swains and Rose Atoll). Administratively, American Samoa is divided into three districts – Eastern District, Western District, and Manu’a District – and the two atolls. The Eastern District consists of the eastern portion of Tutuila island, American Samoa’s largest and most populous island, plus the island of Aunu’u. The Western District consists of the western portion of Tutuila Island. The 2010 census captures the population by district, county and village^29^. The census records 55,519 people living in 77 villages in American Samoa, together with data on age group and gender. The model captured 98% of the population of American Samoa residing in the Eastern and Western districts. Small and remote villages in other areas were excluded. The analysis of household structure is detailed in the Supplementary Information.

The high-resolution geographic locations of households were based on the latest land use data from the American Samoa Department of Commerce^30^. The raw data consists of 16,638 buildings in six major categories: residential, business, commercial, government, religious and school. Duplicated records and other apparent data errors were corrected, and buildings were mapped into villages based on maps of village boundaries. Typical raw records of the buildings are given in the Supplementary Table S1 with fields irrelevant to the study not displayed. The building status can be new, damaged, demolished and destroyed, or not specified (with an empty field).

To model the population evolving over an extended period, the projections of fertility rates, annual number of net migrants from the U.S. Census Bureau’s International Data Base (IDB) were used^35^. The latest estimates and projections for American Samoa were released in June 2012. Although the Population Census 2010 had been released by then, the base population used to develop the estimates and projections was still the 2000 census population. The availability of the 2010 census population only led to the calibrated estimates of the total population and the migration, rather than a fully updated comprehensive set of estimates and projections. The fertility projections were still based on data of registered births in 2004^35^.

The projections of annual deaths are also available in the IDB. However, instead of age-specific mortality rates, the Bureau only released the estimated annual number of deaths, which was highly dependent on both the mortality rates and the population age structure. As the projections of the Bureau were produced based on the 2000 census population, the estimated age structure in 2010 significantly deviated from the true age structure. Therefore, the life table in the statistical yearbook 2015^36^, which was developed based on the population and deaths in 2011, was used in this study. A sample life table for females is given in see Supplementary Table S5.

### Static population

In recent years, sample-free methods have been given increasing attention due to the lesser data requirements and better fitting results to individual and household level data^37^. In this study, an iterative approach proposed by Gargiulo *et al*.^28^ was used to generate the static synthetic population using a sample-free method. The census data was first cross-validated to ensure records from different sources and inconsistencies in the population census were addressed before the generation algorithm. Data errors were present in the land use data from the American Samoa Department of Commerce, which were handled before incorporated into our model. The synthetic population generation process is detailed in the Supplementary Information.

### Population Evolution

Major dynamic processes, including births, deaths, couple formation/separation and migration, were included in the model to allow the population to evolve over decades. The dynamic population was modelled at half-day time steps. The half-day time step setting was used because of possible diurnal variations in the risk of infection, the behaviour of individuals, and prevention/control measures. For example, infection risk can vary between day and night for mosquito-borne infections such as dengue and lymphatic filariasis. The spatial locations and activities of the individuals can also change significantly between day- and night-time, e.g. most people will be at work or school during the day, and at home during the night; and some interventions will only be effective at night, e.g. bed nets.

#### Births and deaths

The number of newborns was calculated based on the projections of the age-specific fertility rates (ASFRs) and the number of females of childbearing age on a daily basis. Newborns were then added into couple households with probabilities based on the female’s age. The sex ratio of newborns was simulated with the latest five-year average (2011–2015, 109 males/100 females), indicating the probability of a newborn being male is 0.522.

The life table in the statistical yearbook 2015^36^ was used to estimate annual deaths in our simulation. An annual 1% decrease in the mortality rate was assumed to account for the observed increased trend in life expectancy^38^. One thing to note is that senior individuals aged over 75 years were classified into the same group in the 2011 life table, implying all individuals more than 75 years were assumed to have the same central rates of mortality. This assumption was expected to underestimate the mortality rates for individuals aged 80+ and to slightly distort the tail of the age structure.

#### Couple formation and separation

The mean age of couple formation (27.3 for males and 26.7 for females) is available from the Population Census 2010, though the distributions about these ages are not provided. Single individuals were assumed to form couples with a fixed probability (${p}_{1}$) each year. A new couple was formed by selecting a single female aged between 18 and 35, and a randomly selected single male (not in the same household as the female) within 3 years of the female’s age. The newly-formed couple was assumed to stay in the male’s current household, e.g., the female will move to the male’s household. An existing couple separates with a fixed small probability (${p}_{2}$). The male and female were assumed to leave the household with an equal probability if they separated. Table 1 summarizes the parameter values assumed in this study.

**Table 1:** Model parameters.

Parameter	Value	Description
*p*_1_	0.15	Annul probability of getting married for single individuals
*p*_2_	0.0001	Annual probability of separation for couples
*p*_3_	0.05	Annual probability of moving to institutionalized households for individuals alone
*p*_4_	0.5	Annual probability of fracture for large households
*s*	10	Threshold for household fracture

*Parameterization: *p*1, assuming 95% individuals will get married before 35; *p*2, observed rare annul divorce cases in the statistical yearbook; *p*3, *p*4 and $s$, chosen to get an expected simulated household size distribution.

#### Movement between households

Movement between households is quite complicated due to the prevalent extended family structure. In reality, a range of events can influence household dynamics, such as reaching adulthood, getting married/divorced, giving birth and being adopted. To reduce the uncertainty related to household dynamics, the types of explicitly modelled events were minimized. Besides the events in Section 4.3.2, other explicitly modelled events include: (i) senior couples or senior individuals living alone will move to live with their children (if any); and (ii) individuals living alone with similar age may move to institutionalized households with a fixed probability (${p}_{3}$) each year. In the 2010 census, the number of institutionalized households is less than 1.59%. ${p}_{3}$ was chosen to allow for a similar percent of institutionalized households in the simulation. A fracturing mechanism was introduced to avoid unexpectedly extremely large households: in each year, large household with size more than a threshold (${\rm{s}}$) had a small probability (${p}_{4}$) to fracture into two separate households. If the fracture happens, the largest family unit (couple and children) other than the householder move to a new household. ${p}_{4}$ and ${\rm{s}}$ together control the percentage of the large households. In our simulation, the number of households with less than ten members is 96.0% in 2010. ${p}_{4}$ and ${\rm{s}}$ were chosen to produce similar results for household sizes in 2020 (after evolving for 10 years).

#### Migration

Migration is a key aspect of demographic change in American Samoa, as the number of migrants is large compared to the total population. In 2015, the loss of the population due to migration was about 4.4% of the total population^36^. The large number of emigrants (mostly young adults going abroad to work or study) has led to a distorted age structure. In the 2010 census population, the proportion of people aged 25–34 years was found to be unexpectedly low in comparison to those aged 35–44 years (see Fig. 1(a)). The projected annual number of migrants from 2010 to 2050 in the IDB were used in our simulation. Individuals aged 25–34 years (primarily) and 35–39 years (with decreased probability) were randomly selected, and the model assumed that the whole household (partner and children) would emigrate together with the chosen individual.

### Data availability

The datasets generated during and/or analysed during the current study are available from the corresponding author on reasonable request.

### Significance Statement

This research built a statistically realistic synthetic population with high-resolution household locations and demographic evolution over an extended period for agent-based modelling of infectious diseases based on the latest population census, land use data and survey results. The statistical characteristics of the simulated population were compared to the projections from the United States Census Bureau to examine the reliability of the base population and population dynamics. Some inconsistencies between the simulations and Bureau projections were found. The synthetic population can serve as the base population for modelling a variety of infectious diseases, especially for diseases with long incubation and long infectious periods, or diseases with highly focal transmission hotspots. It also offers an alternative for investigating demographic evolution.

## Electronic supplementary material

Supplementary Information
