# Supplementary material for: A Synthetic Population for Modelling the Dynamics of Infectious Disease Transmission in American Samoa
Source: Sci Rep. 2017 Dec 1;7:16725. doi: 10.1038/s41598-017-17093-8 (PMC5711879; doi:10.1038/s41598-017-17093-8)
Supplement: Supplementary file 1 — Supplementary Information [file 41598_2017_17093_MOESM1_ESM.pdf]

Title: A Synthetic Population for Modelling the Dynamics of Infectious Disease Transmission in American Samoa

Author Affiliation:

Zhijing Xu[1], Kathryn Glass[1], Colleen Lau[1], Nicholas Geard[2,3], Patricia Graves[4], Archie Clements[1]

[1] Research School of Population Health, ANU

[2] School of Computing and Information Systems, University of Melbourne

[3] Melbourne School of Population and Global Health, University of Melbourne

[4] College of Public Health, Medical and Veterinary Sciences, Division of Tropical Health and Medicine, James Cook University

Corresponding Author:

Zhijing Xu

Building 62, Mills Road, The Australian National University, Acton, 2601

Tel: +61 02 6125 6803

Email: sting.xu@anu.edu.au

Key words: synthetic population, agent-based modelling, infectious disease dynamics, demographic change, spatial heterogeneity

Supplementary Information

Data and Materials

In 2010 census, American Samoa had 10,963 houses, of which 88% were occupied (9,688) and 12% (1,275) were vacant, with occupancy data available by village. Family households have traditionally accounted for a large proportion of all households in American Samoa, in which the extended family unit is the most important familial structure [40]. Historically, household sizes are large due to cultural conceptions of the family where multiple generations cohabit [40]. In 2000, 93% of 9,349 households in American Samoa were family households; of these, 40% contained seven or more persons [41], similar to the survey results in 2010 [18,27], in which 48.9% of participants lived in the households with more than seven members.

The 2010 survey included 807 adults (aged 18 to 87 years, 52.4% males) from 659 households in 55 villages on all five inhabited islands of American Samoa, in which sampling was designed to provide a representative sample of the adult population in relation to age, sex, and geographic distribution [18,27]. From the 2010 LF seroprevalence survey (807 participants) [18,27], the household size in American Samoa follows a Gaussian distribution with parameters $\mu = 6.06$ and $\sigma = 2.86$ ($R^2 = 0.952$, red dashed line in Figure 3(a)). However, census records show that the average household size has continuously decreased from 7.2 in 1980 to 5.6 in 2010. These data were combined to determine the household size distribution by assuming that the mean household size (μ) in each village was equal to its population divided by the number of housing units. With no further data available, the standard deviations of household size in all the villages were assumed to be $\sigma = 2.86$.

Annual total live births showed a decreasing trend from 1,720 in 2005 to 1,096 in 2015 [36], while annual deaths were relatively stable, varying between 250 and 330 in most years. Consequently, population natural growth, defined as total births minus deaths, has been slowly decreasing in recent years. Additionally, due to the high levels of net emigration, as indicated by the data of international arrivals and departures in the statistical yearbook, the total population in American Samoa slightly decreased in recent years. In 2015, the natural growth was 782 and the loss of the population due to emigration was 2,449, indicating a net loss of 1,667 persons.

The latest projections of 2010-2050 ASFRs (Supplementary Table S4) are produced based on the 2000 census population and registered births in 2004, and are available through the IDB [35]. Supplementary Fig. S3 indicates that the Bureau assumed exponentially decreasing fertility rates for each five-year age group between 15 and 44. The ASFRs for age group 45-49 was projected to remain stable at a low level throughout the 40-year period.

Synthetic Population Generation

There are two approaches for generating synthetic populations. Sample-based methods use a sample of the population from a larger region, and apply synthetic reconstruction [42] and combinatorial optimization [43] to generate a synthetic population. The quality of the synthetic population for sample-based methods is highly dependent on the quality of the initial sample [37]. In the United States, the sample is generated from a larger region (Public Use Microdata Area, PUMA) and is known as a Public Use Microdata Sample (PUMS) [44]. A PUMS record is essentially a complete census record with identifying information such as names and addresses removed. For regions such as American Samoa, a high quality sample of the population is not available and it is difficult to generate synthetic populations based on sample-based methods that match both the households' and individuals' joint distributions [45].

Before the implementation of the synthetic population generation algorithm, the census data were first cross-validated to ensure records from different sources were consistent. For example, both the data of village population by age group (Supplementary Table S2) and the population data by age (Supplementary Table S3) provide an estimate of the total population in a specific age group. For any age group, the population numbers from these two data sources should be consistent, and inconsistencies must be addressed before producing the synthetic population. For example, the population in age group 20-24 was recorded as 3,922 in the population data for each village, and as 3,890 in the total population. In this study, the base population was the village population by age group and other data sources were used to calibrate the base population. Five types of households were modelled according to the population census: (i) couple household; (ii) female householder with no husband; (iii) male householder with no wife; (iv) householder living alone; and (v) householder living with non-relatives. The synthetic population was generated as follows (see Gargiulo et al. (28) for further details):

a. The population in each village by age group was constructed, assuming uniform distributions in each age group;

b. The synthetic population in each village was further categorised by gender, according to the sex ratio by age group;
c. Housing units in each village were developed as follows:
    i. The frequency of households with specified sizes were calculated using the Gaussian distribution obtained from fitting to the baseline data and the total number of households in each village;
    ii. If the total capacity of the built households was larger (or smaller) than the population, households were randomly chosen to decrease (or increase) their capacity to a randomly selected size from the Gaussian distribution until the total capacity of the built households matched the population. In this step, 20% of randomly selected small household units were merged to reflect the presence of extremely large households in the population of American Samoa.
d. The population in each village was allocated into the households as follows:
    i. The maximum number of couple households was calculated according to the number of males and females aged over 18;
    ii. A random household type for each household was selected according to the recorded frequency of each type, considering the constraints due to the number of males and females;
    iii. Each household was assigned a householder and a partner (if applicable), with the age difference between partners minimized;
    iv. Each child aged less than 18 was allocated a household, considering constraints due to the age of mother (or householder), e.g., the age difference with the mother should be more than 18;
    v. Each male not in a household after step (iv) was allocated into a random appropriate household with a vacancy;
    vi. Each female not in a household after step (iv) was allocated into a random appropriate household with a vacancy.

Some errors were present in the land use data from the American Samoa Department of Commerce. The most common was that the integral parts of the building were recorded as several independent buildings in the GIS data. Instinctively, the integral parts of a building should be very close to each other and there should be a main building with significantly larger area than the others. In our algorithm, these errors were handled as follows: buildings with distances between them of less than one meter were treated as parts of the same building;

Furthermore, if the straight-line distance between two buildings was less than 20m and the area of one building was less than 25% of the other, they were treated as parts of the same building. When merging buildings, very large buildings with areas greater than 1,000 sqm were not allowed. All residential buildings with unreasonable size (areas less than 30 or more than 1,000 sqm) were removed if there were still any left after the merging step. In some villages, the number of buildings was less than the number of housing units, possibly because new houses had been built since the building database was created. Residential buildings were artificially created and added to these villages as follows: (i) two buildings more than 20m apart were randomly selected; (ii) a new building with area equal to the average of the two buildings was added at the midpoint of the two buildings. Each household was given the location of a residential building in American Samoa.

References

40 Holmes, L. D. Factors contributing to the cultural stability of Samoa. *Anthropological Quarterly*, 188-197 (1980).

41 *Demographic Baseline Report of U.S. Territories and Counties Adjacent to Coral Reef Habitats - American Samoa*, https://www.coris.noaa.gov/activities/coral_demographics/

42 Arentze, T., Timmermans, H. & Hofman, F. Creating synthetic household populations: problems and approach. *Transportation Research Record: Journal of the Transportation Research Board*, 85-91 (2007).

43 Voas, D. & Williamson, P. An evaluation of the combinatorial optimisation approach to the creation of synthetic microdata. *Population, Space and Place* **6**, 349-366 (2000).

44 Adiga, A. *et al.* Generating a synthetic population of the United States. Report No. NDSSL Technical Report 15-009, (Virginia Bioinformatics Institute, 2015).

45 Barthelemy, J. & Toint, P. L. Synthetic Population Generation Without a Sample. *Transportation Science* **47**, 266-279, doi:10.1287/trsc.1120.0408 (2012).

Tables

Supplementary Table S1. Typical building records

BLD_ID	AREA (sqm)	BLD_CATEG	STATUS	x (meters)	y (meters)
16376	296.400	Residential		527692.92914100000	8412260.36398000000
16361	98.463	Residential	Destroyed	535449.88927000000	8424318.21739000000
13837	418.879	Government		534875.81945400000	8420674.61716000000
10358	556.266	School		523246.43049100000	8414822.04605000000
10203	898.582	Commercial		523869.29740600000	8414456.90519000000
13373	48.657	Commercial	New	529387.98800500000	8416530.39197000000
3091	123.853	Residential	Demolished	533269.94034900000	8421580.67689000000
9968	274.421	Commercial	Damaged	532044.02066900000	8421993.94223000000

Supplementary Table S2. Village population by age group: 2010 (sample)

Place	Total	Males	Females	Under 5	5-9	10-14	15-19	20-24	25-34	35-44	45-54	55-59	60-64	65-74	75-84	85+	Median Age
Total	55,519	28,170	27,349	6,611	6,535	6,279	6,297	3,922	6,835	7,196	6,022	2,067	1,480	1,616	546	113	22.4
Aasu	494	251	243	65	69	48	61	37	55	62	54	15	11	15	1	1	20.6
Afao	182	91	91	25	21	32	18	11	32	17	15	5	1	4	1	0	18.3
Afono	524	271	253	54	58	78	75	42	40	73	54	18	11	14	5	2	19.7
Agugulu	51	29	22	7	9	3	6	3	5	9	5	4	0	0	0	0	20.5
Alao	495	243	252	57	44	59	79	36	49	70	57	14	10	14	5	1	20.8
Alega	54	26	28	6	3	4	5	4	7	6	5	2	6	4	1	1	29.0
Alofau	646	329	317	79	62	54	81	46	78	114	76	23	13	15	4	1	25.1
Amaluia	162	82	80	15	18	20	20	10	16	15	26	11	4	6	1	0	24.0
Amanave	250	122	128	29	27	28	33	21	26	31	36	6	7	5	1	0	22.0
Amaua	96	43	53	9	3	7	14	5	13	14	19	3	3	5	1	0	32.0
Amouli	920	468	452	111	120	119	100	52	103	123	103	29	18	29	8	5	20.7
Anua	18	10	8	2	2	4	0	0	1	4	4	0	0	0	1	0	3.3.5
Aoa	855	426	429	132	119	88	91	45	112	103	89	36	12	18	9	1	19.8
Aoloau	615	314	301	76	67	58	68	55	77	83	65	16	22	15	11	2	22.9
Asili	224	120	104	31	32	28	24	15	18	26	24	6	10	6	4	0	19.3
Atu'u	359	197	162	25	43	45	50	24	38	53	44	12	14	5	5	1	22.9
Aua	2,077	1,097	980	252	255	239	230	153	261	254	239	78	46	49	15	6	21.8
Auasi	113	61	52	10	9	18	16	7	15	15	8	5	2	6	2	0	23.5
Auma	254	137	117	27	23	34	40	11	24	34	36	12	7	5	1	0	21.5
Aumi	186	89	97	32	22	23	20	13	15	19	21	5	4	10	1	1	19.2
Aunu'u	436	221	215	57	58	49	49	30	57	41	52	16	10	10	6	1	20.6

Supplementary Table S3. Population by age: 2010

Age Group	2010	Males	Females
Total	55,519	28,170	27349
0-4	6,611	3,417	3194
5-9	6,535	3,470	3065
10-14	6,279	3,214	3065
15-19	6,297	3,218	3079
20-24	3,890	1,944	1946
25-29	3,325	1,670	1655
30-34	3,506	1,724	1782
35-39	3,604	1,844	1760
40-44	3,602	1,795	1807
45-49	3,387	1,673	1714
50-54	2,678	1,336	1342
55-59	2057	1,014	1043
60-64	1481	754	727
65-69	957	500	457
70-74	653	323	330
75+	657	274	383

Supplementary Table S4. Estimates and Projections of ASFR from the Bureau (sample) (32)

Year	15-19	20-24	25-29	30-34	35-39	40-44	45-49	Total Fertility Rate
2000	54.60	196.30	241.60	178.60	101.20	26.70	0.00	4.00
2010	34.20	139.10	212.50	151.40	80.70	23.60	2.30	3.22
2011	33.20	136.10	208.70	149.20	79.00	23.20	2.30	3.16
2012	32.30	133.10	205.00	147.00	77.30	22.70	2.30	3.10
2013	31.30	130.10	201.30	144.80	75.60	22.30	2.30	3.04
2014	30.30	127.00	197.50	142.70	73.90	21.90	2.30	2.98
2015	29.40	124.00	193.80	140.50	72.30	21.50	2.30	2.92
2016	28.60	121.60	190.80	138.70	70.90	21.10	2.30	2.87
2017	27.90	119.20	187.90	137.00	69.60	20.80	2.30	2.82
2018	27.10	116.80	184.90	135.20	68.20	20.50	2.20	2.78
2019	26.30	114.40	181.90	133.50	66.90	20.10	2.20	2.73
2020	25.60	112.00	178.90	131.70	65.50	19.80	2.20	2.68
2021	25.00	110.10	176.50	130.30	64.40	19.50	2.20	2.64
2022	24.40	108.20	174.20	129.00	63.40	19.30	2.20	2.60
2023	23.80	106.30	171.80	127.60	62.30	19.00	2.20	2.57
2024	23.20	104.40	169.50	126.20	61.20	18.80	2.20	2.53
2025	22.60	102.50	167.10	124.80	60.20	18.50	2.20	2.49
2026	22.10	101.10	165.40	123.80	59.40	18.30	2.20	2.46
2027	21.70	99.70	163.60	122.80	58.60	18.10	2.20	2.43
2028	21.20	98.30	161.90	121.70	57.80	17.90	2.20	2.41
2029	20.80	96.90	160.10	120.70	57.00	17.70	2.20	2.38
2030	20.30	95.50	158.40	119.70	56.20	17.50	2.10	2.35

*As of June 2017

Supplementary Table S5. American Samoa Life Table (Female)

Age	Average death	2011 population	Mx	qx	Lx	Ex
< 1	4	707	0.005658	0.005629	100000.0	77.762907
1-4	1	2,550	0.000392	0.001567	99437.1	77.202550
5-9	1*	3,032	0.000330	0.000000	99281.2	73.320608
10-14	1	3,032	0.000330	0.001648	99281.2	68.320608
15-19	1*	3,132	0.000106	0.000532	99117.6	63.429251
20-24	1	2,064	0.000323	0.001613	99064.9	58.461687
25-29	2	1,563	0.001280	0.006380	98905.1	53.552118
30-34	4	1,687	0.002173	0.010808	98274.1	48.879899
35-39	2	1,715	0.000972	0.004848	97212.0	44.386634
40-44	4	1,773	0.002255	0.011214	96740.7	39.590697
45-49	7	1,746	0.003819	0.018916	95655.8	35.011350
50-54	8	1,413	0.005898	0.029060	93846.4	30.638193
55-59	11	1,095	0.010351	0.050449	91119.2	26.480374
60-64	11	778	0.014131	0.068243	86522.3	22.754442
65-69	11	495	0.021541	0.102201	80617.8	19.237895
70-74	12	333	0.035079	0.161251	72378.5	16.143262
75+	30	413	0.072642	1.000000	60707.4	13.766212

*values 0 is adjusted to be 1

Notes:

Mx - central rate of mortality;

qx - the probability that someone aged *x* will die before reaching age *x* + 1;

Lx - the number of people who survive to age *x*;

Ex – the remaining life expectancy of someone age *x*;

Suppl. Figures

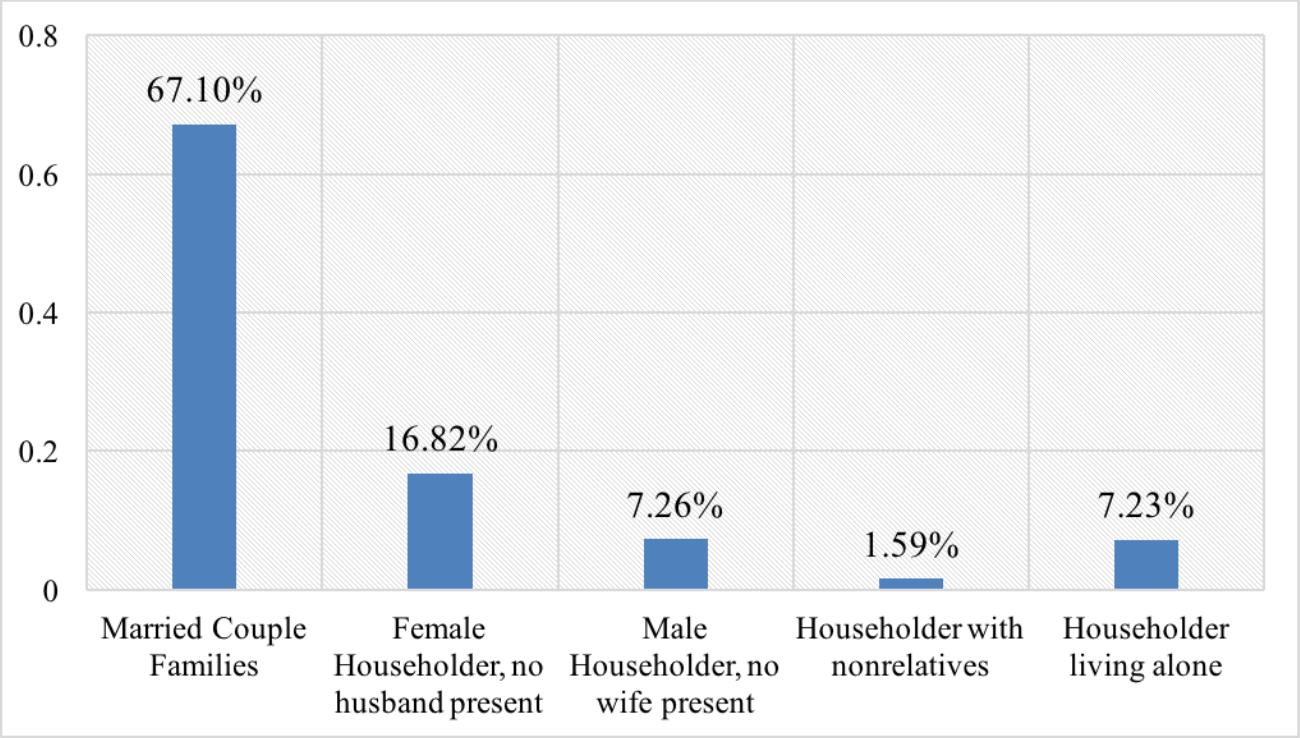

Supplementary Figure S1. Household by type in the Population Census 2010. Total number of households is 9,688. Children can only be present in the first three household types.

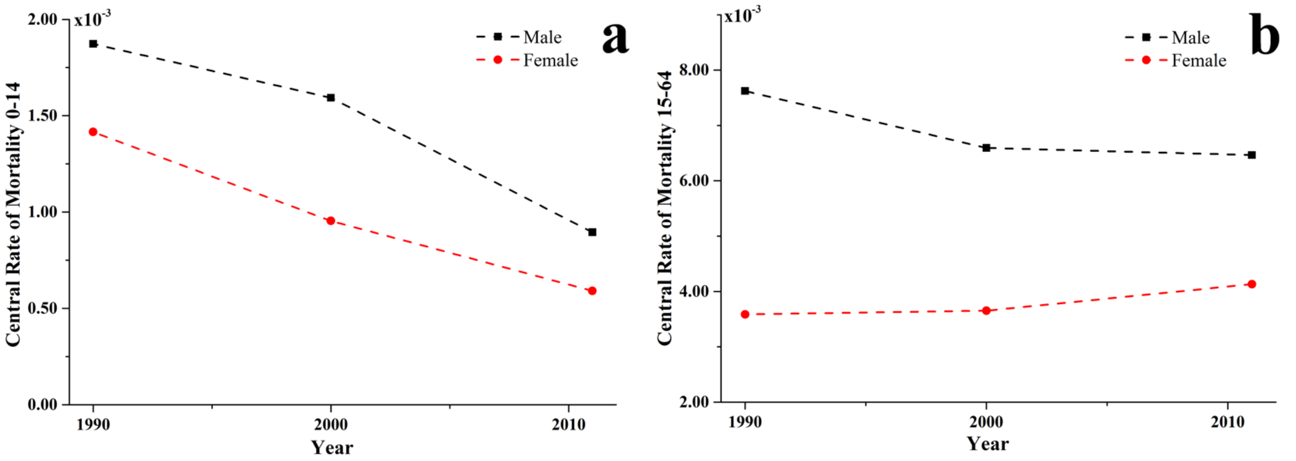

Supplementary Figure S2. Central rates of mortality (Mx(s)) 1990-2011 by age group: (a) 0-14; (b) 15-64. Mx(s) were calculated based on the life table in the Population Census 1990, 2000 and 2011. The mortality rate was calculated based on the life table in the 2000 census population and the statistical yearbook 2009, 2015.

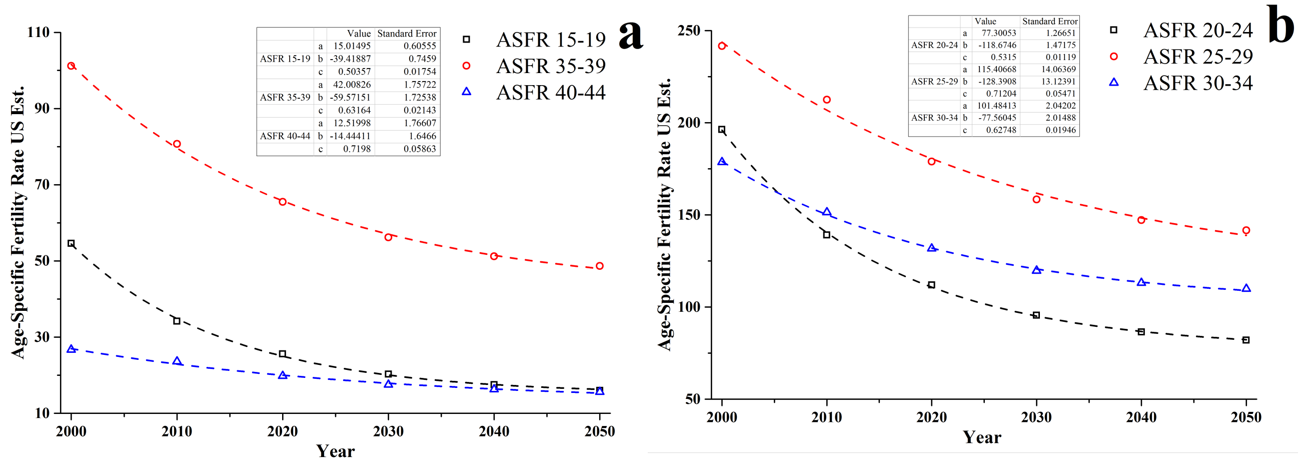

Supplementary Figure S3. Age-specific fertility rate 2010-2050 from the Bureau. The dashed lines are exponential fittings to the projections with the function $y = a - b \times c^x$ with parameters in the embedded tables.
